# Treating phenotype as given: a simple resampling method for genome-wide association studies

**DOI:** 10.1186/1753-6561-5-S9-S60

**Published:** 2011-11-29

**Authors:** Kai Wang, Jian Huang

**Affiliations:** 1 Department of Biostatistics, University of Iowa, Iowa City, IA 52242, USA; 2 Department of Statistics and Actuarial Sciences, University of Iowa, Iowa City, IA 52242, USA

## Abstract

Significance of genetic association to a marker has been traditionally evaluated through statistics that are standardized such that their null distributions conform to some known ones. Distributional assumptions are often required in this standardization procedure. Based on the observation that the phenotype remains the same regardless of the marker being investigated, we propose a simple statistic that does not need such standardization. We propose a resampling procedure to assess this statistic’s genome-wide significance. This method has been applied to replicate 2 of the Genetic Analysis Workshop 17 simulated data on unrelated individuals in an attempt to map phenotype Q2. However, none of the selected SNPs are in genes that are disease-causing. This may be due to the weak effect that each genetic factor has on Q2.

## Background

The traditional approach to hypothesis testing is to construct a test statistic whose exact or approximate distribution under the null hypothesis is known. Alternative methods for assessing significance of a test statistic include resampling techniques, such as the bootstrap and permutation. These methods dominate modern genetic association studies.

Statistics used in these methods typically make use of a standardization factor, for instance, the standard error of the observed genetic effect size. Different model assumptions result in different standardization factors. For instance, in a case-control genetic association study, we are interested in testing whether the difference in the frequency of a reference allele between case subjects and control subjects is 0. The popular allelic test and the Cochran-Armitage test for trend are based on two different estimates of the variance of this difference. The estimate for the allelic test requires Hardy-Weinberg equilibrium in the combined case-control population, whereas the estimate for the Cochran-Armitage test does not. The properties of these two tests are rather different [1].

It is a brilliant idea to construct a test statistic through standardization. It makes it possible to assess the significance of the observed effect size with respect to a reference distribution. However, in genome-wide association studies there are typically tens of thousands of markers. Presumably most of these markers are under the null hypothesis (i.e., they are not associated). The presence of these null markers provides a natural reference distribution. Standardizing the effect size in this setting appears to be unnecessary and awkward.

We propose a novel resampling approach that aims to directly assess the significance of genetic effect size. The null distribution generated from this method is for a randomly selected single-nucleotide polymorphism (SNP). Hence it naturally addresses the genome-wide significance of genetic effect size at a SNP and obviates the issue of multiple testing. To define the genetic effect size, we observe that the phenotype remains the same in a genome scan. Only the testing locations are different. This observation leads to a simple representation of the genetic effect size. This approach is demonstrated by means of a genome-wide association study on phenotype Q2 in replicate 2 of the Genetic Analysis Workshop 17 (GAW17) simulated data on unrelated subjects.

## Methods

Let *n* denote the number of individuals in a study. Let *y_i_* denote the measure of a quantitative phenotype *y* on individual *i*. The score of the genotype at a SNP is denoted by *g*. Assuming that the two alleles of the SNP are denoted by A and B, we set *g* = 0 for genotype AA, *g* = 1 for genotype AB, and *g* = 2 for genotype BB. The genotype score on individual *i* is denoted by *g_i_*.

To identify SNPs associated with phenotype *y*, we consider a regression in which the response variable is the genotype score *g* and the independent variable is the phenotype *y*. The (partial) regression sum of squares for *g* equals:(1)

where  and  are the sample means of *g_i_* and *y_i_*, respectively. Because the denominator in expression (1) remains constant in a genome scan, a natural measure of association would be:(2)

Note that in a case-control study  is proportional to the difference in the frequency of allele B between case subjects and control subjects. If there are *p* covariates *x_j_*, *j* = 1, …, *p*, then phenotype *y_i_* is replaced by the residual  from the following linear regression:(3)

where *x_ij_* is the value of *x_j_* for individual *i*. Similarly, genotype *g* is replaced by the residual g* of the regression of *g* over *x_j_*, *j* = 1, …, *p*.

We use the statistic:(4)

to measure the strength of association, where n* is the number of subjects whose genotype is nonmissing at the SNP being investigated. The sample mean of  is 0, so it is dropped from the definition of *S*. So is the sample mean of . The purpose of using n* is to make the statistic *S* on the same scale because n* is expected to vary across the genome.

In comparison, the usual method for detecting association would consider the following regression:(5)

The least-squares estimate of *β*_1_, denoted , is:(6)

The statistic for testing for association is:(7)

where  is the standard error of . This procedure is equivalent to testing whether the coefficient of *g* is 0 in a multiple linear regression that includes *x_i_*, …, *x_p_* as covariates and *y* as the response. Because the total sum of squares for  is fixed in a genome scan, there is a monotonic relationship between the regression sum of squares for Eq. (5) and the statistic *T*.

We propose the following resampling procedure to evaluate the genome-wide significance of *S*: (1) Compute residuals  and residuals  from regression (3). (2) Randomly select a SNP from a set of SNPs that are under the null hypothesis. This null set can be determined by using a histogram of *p*-value [2]. (3) Permute residuals  (or, equivalently, permute residuals ). (4) Compute the statistic *S* for the randomly selected marker. (5) Repeat steps 2–4 the desired number of times, say *K*. (6) Let *S*^(^*^k^*^)^ denote the value of statistic *S* in the *k*th iteration. Suppose that the value of statistic *S* is *s* for the observed data. Its *p*-value is computed as:(8)

where *I*(·) is the indicator function satisfying *I*(True) = 1 and *I*(False) = 0.

Because the SNP selected in each iteration is random, the significance obtained in this way is genome-wide.

## Results

**Figure 1:**
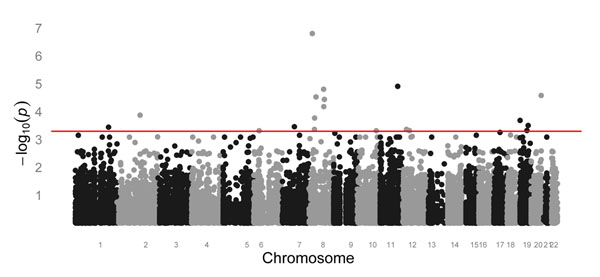
**Genome-wide plot of *p*-values for the regular statistic *T***. The horizontal line corresponds to *p* = 5 × 10^−4^.

We analyze phenotype Q2 in replicate 2. There are 24,487 SNPs and 697 individuals. Residuals  are computed from regression (3) with regressors Sex, Age, and Smoke. The residuals  are computed in the same way. Genome-wide *p*-values from the *t*-distribution with 1 degree of freedom for statistic *T* are presented in Figure 1. There are 19 SNPs with a *p*-value less than 5 × 10^−4^ (Table 1).

**Table 1:** Selected SNPs based on *p*-values of statistic *T* (*p* < 5 × 10^−4^)

					*p*-value	
					
Chromosome	Gene	SNP	Minor allele frequency	Base-pair position	Statistic *T*	Statistic *S*
1	*NEK7*	C1S8842	0.001435	196488886	3.55 × 10^−4^	1.53 × 10^−1^
2	*R3HDM1*	C2S3088	0.002152	136095600	1.32 × 10^−4^	1.18 × 10^−1^
6	*BAT2*	C6S2242	0.008608	31712334	4.78 × 10^−4^	5.59 × 10^−2^
7	*MLXIPL*	C7S1785	0.017217	72658237	3.43 × 10^−4^	1.59 × 10^−2^
8	*NRG1*	C8S1224	0.001435	32625157	4.28 × 10^−4^	1.56 × 10^−1^
	*RBM13*	C8S1319	0.005022	33475616	1.67 × 10^−4^	4.63 × 10^−2^
	*ANK1*	C8S1496	0.005022	41638505	2.92 × 10^−5^	6.35 × 10^−2^
	*CA3*	C8S2634	0.029412	86539249	1.55 × 10^−5^	5.59 × 10^−3^
	*CNGB3*	C8S2646	0.105452	87748419	6.52 × 10^−5^	5.72 × 10^−4^
	*NBN*	C8S2710	0.022238	91064195	3.58 × 10^−5^	8.67 × 10^−3^
	*LPL*	C8S442	0.015782	19849988	1.54 × 10^−7^	1.30 × 10^−2^
10	*DUSP5*	C10S5537	0.133429	112256813	4.77 × 10^−4^	7.31 × 10^−4^
11	*PDGFD*	C11S5292	0.008608	103413068	1.21 × 10^−5^	3.89 × 10^−2^
12	*SLCO1A2*	C12S1083	0.001435	21319511	4.28 × 10^−4^	1.56 × 10^−1^
	*LRRK2*	C12S1884	0.023673	38978429	4.72 × 10^−4^	2.24 × 10^−2^
19	*C19ORF6*	C19S56	0.018651	963559	2.04 × 10^−4^	1.96 × 10^−2^
	*CAPNS1*	C19S3468	0.012912	41325704	4.69 × 10^−4^	3.69 × 10^−2^
	*PSG9*	C19S4603	0.030129	48465412	3.10 × 10^−4^	7.17 × 10^−3^
20	*PRIC285*	C20S2223	0.003587	61666107	2.59 × 10^−5^	6.27 × 10^−2^

**Figure 2:**
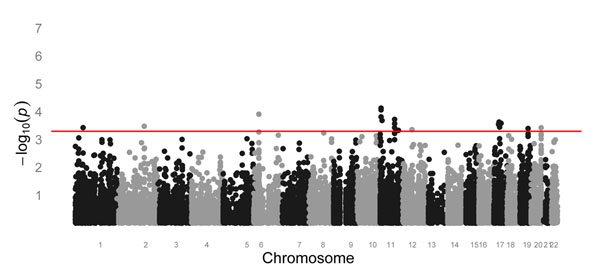
**Genome-wide plot of resampling *p*-values for the proposed statistic *S.*** The horizontal line corresponds to *p* = 5 × 10^−4^.

The reference distribution for statistic *S* is obtained in the way described in the previous section. The value of *K* is set at 10,000,000. The histogram of *p*-values for parameter *β*_1_ in regression (5) shows no apparent deviation from uniform distribution (data not shown). Thus all SNPs are used to determine the reference distribution for statistic *S*. *P*-values obtained from the resampling procedure are presented in Figure 2. Eighteen SNPs have a *p*-value that is less than 5 × 10^−4^. None of these SNPs are close to the 19 SNPs that have *p*-values less than 5 × 10^−4^ for statistic *T*, except for C20S2223 on chromosome 20 (base-pair position 61666107; *p* = 2.59 × 10^−5^ for statistic *T*; resampling *p* = 6.27 × 10^−2^ for statistic *S*). C20S2223 is located in gene *PRIC285* identified by statistic *S*.

## Discussion and conclusions

Significance of genetic association at a SNP has been traditionally evaluated through a standardized test statistic such that its significance can be evaluated through a known distribution. For instance, in Eq. (7) the standard error of  serves as a standardization factor. Its *p*-value is assessed through a *t*-distribution or a standard normal distribution. This strategy works beautifully in general but is awkward in the context of genome-wide association studies. The presence of abundant unassociated SNPs provides a natural reference distribution that is pertinent to the data. We have proposed a simple statistic *S* and a resampling method for generating its reference distribution. Although a distributional assumption is necessary to compute the standard error of , it is not required in our method. More important, the resampling procedure has a built-in mechanism for handling genome-wide significance because the reference distribution is the distribution of an arbitrary SNP under the null hypothesis that there is no association. Application to the GAW17 simulated data on unrelated individuals (replicate 2) revealed several susceptible genes for phenotype Q2 that can be used as candidates for further investigation.

The essence of the proposed method is the existence of a large number of similar features—SNPs in the current context. The basic principle is applicable to other situations that involve a large amount of similar features, for instance, gene expression levels in gene expression studies.

We have focused on a continuous trait. As alluded to earlier, for a dichotomous trait, if there are no covariates, then the statistic *S* can be defined as the difference in frequency of the reference allele between the two phenotype groups. More generally, one can use a proportional odds model with genotype as the response and phenotype as the predictor. A proportional odds model is appealing for dealing with covariates and both continuous and dichotomous traits. We are actively investigating this possibility.

The proposed method is resampling based and requires intensive resampling. However, computation for each sample is fast. The overall computation time for *K* = 10,000,000 resampling iterations took less than two days on a computer running the Windows Vista operating system. The iteration coverage is about 408 per SNP (≈ *K*/24,487 SNPs). We have also used *K* = 100,000; the simulated *p*-value remains almost the same. The resampling procedure needs to be done only once, regardless of the number of SNPs.

We are investigating the utility of our approach in other association study settings, such as gene-gene interaction and population stratification.

We had no knowledge of the GAW17 disease-generating model when this analysis was conducted. It turns out that none of the genes in Table 1 and Table 2 are disease causing. This may be due to the weak effect that each genetic factor has on Q2.

**Table 2:** Selected SNPs based on resampling *p*-values of statistic *S* (*p* < 5 × 10^−4^)

					*p*-value	
					
Chromosome	Gene	SNP	Minor allele frequency	Base-pair position	Statistic *T*	Statistic *S*
1	*LOC100133124*	C1S2906	0.32	46294146	3.99 × 10^−3^	3.70 × 10^−4^
2	*LY75*	C2S4258	0.45	160419168	1.25 × 10^−2^	3.31 × 10^−4^
6	*HLA-A*	C6S1568	0.30	30020831	8.07 × 10^−3^	1.21 × 10^−4^
11	*OR52H1*	C11S662	0.31	5522627	1.31 × 10^−3^	7.35 × 10^−5^
		C11S665	0.32	5522688	3.15 × 10^−3^	1.52 × 10^−4^
		C11S675	0.32	5523065	1.59 × 10^−3^	8.52 × 10^−5^
	*GALNTL4*	C11S1338	0.43	11310922	3.25 × 10^−3^	2.04 × 10^−4^
	*SYTL2*	C11S4881	0.38	85113378	2.36 × 10^−3^	1.87 × 10^−4^
		C11S4893	0.31	85114042	5.41 × 10^−3^	3.70 × 10^−4^
		C11S4949	0.48	85134351	6.79 × 10^−3^	2.66 × 10^−4^
	*LOC100128794*	C11S5677	0.30	107889417	8.72 × 10^−3^	4.65 × 10^−4^
12	*OR6C76*	C12S3054	0.26	54106388	4.77 × 10^−3^	4.37 × 10^−4^
17	*RHOT1*	C17S1743	0.35	27558083	6.77 × 10^−3^	2.81 × 10^−4^
	*SLFN13*	C17S1964	0.42	30792467	9.33 × 10^−3^	2.33 × 10^−4^
	*PIP4K2B*	C17S2376	0.40	34180257	7.62 × 10^−3^	3.55 × 10^−4^
	*CDC27*	C17S3299	0.46	42589359	2.68 × 10^−3^	2.58 × 10^−4^
19	*PSG3*	C19S4193	0.34	47935058	1.73 × 10^−2^	4.00 × 10^−4^
20	*PRIC285*	C20S2305	0.22	61671020	5.23 × 10^−3^	3.72 × 10^−4^

## Competing interests

The authors declare that there are no competing interests.

## Authors’ contributions

KW conceived of the study and participated in its design and coordination and helped to draft the manuscript. JH helped to draft the manuscript. All authors read and approved the final manuscript.
